# Bias in the use of randomized trials for carotid stenosis management

**DOI:** 10.1007/s00772-015-0036-3

**Published:** 2015-06-23

**Authors:** A.L. Abbott

**Affiliations:** School of Public Health and Preventive Medicine, Monash University, 99 Commercial Road, 3004 Melbourne, Australia

**Keywords:** Randomized trial, Bias, Carotid stenosis, Stroke prevention, Evidence, Randomisierte Studie, Bias, Karotisstenose, Schlaganfallprävention, Evidenz

## Abstract

**Background:**

Carotid artery procedures, such as surgery and stenting, although associated with significant risks and costs, are often recommended in guidelines which cite12- to 34-year-old randomized trial evidence of benefit; however, these recommendations exist although there is no evidence these procedures benefit patients who receive only current optimal medical treatment  (encouragement of a healthy lifestyle and appropriate use of medication).

**Objective:**

To examine whether bias exists in the use of randomized trial evidence and its impact on guideline recommendations.

**Material and methods:**

Examples of how bias underpins endorsement of carotid procedures for patients with asymptomatic or symptomatic carotid stenosis were sought from available literature. .

**Results:**

Many forms of procedural bias were identified involving the need for randomized trials, and their design and interpretation. Fundamental problems included failure to first adequately measure outcomes with non-invasive treatment alone, lack of appreciation of quality non-randomized trial measurements of risk in determining need for randomized trials and their applicability in routine practice, poor randomized trial methods with biased comparisons, inaccurate definitions of target populations, confusion of efficacy and safety outcomes, too much reliance on statistical rather than clinical significance and biased use of terminology to make procedures sound more effective.

**Conclusion:**

Procedural bias in design and interpretation of randomized trials has resulted in widespread loss of understanding of how to optimize outcomes in patients with carotid artery stenosis. Current guidelines reflect the cumulative impact of this bias and are an excellent starting point for efforts to improve prevention of stroke and other vascular disease complications; however, there is also need for clinicians, policy makers, health service funding bodies, educators and the general public to assist.

## Abstract (de)

**Hintergrund:**

Häufig werden in Leitlinien mit dem Hinweis auf Evidenz aus randomisierten Studien Interventionen an der A. carotis empfohlen, etwa Operationen oder Stenteinlagen, obwohl diese mit erheblichen Risiken und Kosten einhergehen. Und solche Empfehlungen gibt es auch ohne Evidenz dafür, dass Patienten, die rein internistisch optimal behandelt werden, von diesen Interventionen profitieren.

**Ziel:**

Überprüft werden sollte ein mögliches Bias bei der Verwendung von Evidenz aus randomisierten Studien und sein Einfluss auf Leitlinienempfehlungen.

**Material und Methoden:**

Beispiele werden vorgestellt, an denen sich zeigen lässt, wie ein Bias die Empfehlung für Interventionen bei asymptomatischer bzw. symptomatischer Karotisstenose untermauert.

**Ergebnisse:**

Viele Formen von prozeduralem Bias wurden identifiziert, sie bedingen den Bedarf für randomisierte Studien, Design und Interpretation. Grundlegende Probleme waren u. a.: Es wurde nicht zunächst das Outcome nach nichtinvasiver Therapie allein adäquat ausgewertet, bei der Festlegung der Notwendigkeit für randomisierte Studien und bei der Anwendbarkeit in der Routine wurden qualitativ hochwertige, nichtrandomisierte Studien zum Risiko nicht berücksichtigt, verwendet wurden mangelhafte Untersuchungsmethoden mit voreingenommenen Vergleichen, Zielkollektive wurden nicht richtig definiert, Outcomes zur Effektivität wurden mit denen zur Sicherheit verwechselt, zu viel wurde entschieden aufgrund der statistischen und zu wenig aufgrund der klinischen Bedeutung, und eine Bias-basierte Verwendung der Terminologie ließ Interventionen effektiver scheinen als sie waren.

**Fazit:**

Ein prozedurales Bias beim Design und bei der Interpretation von randomisierten Studien hat zu einem weit verbreiteten Verlust geführt: Es wird nicht mehr verstanden, wie sich das Outcome bei Karotisstenose optimieren lässt. Der kumulative Einfluss dieses Bias spiegelt sich wider in aktuellen Leitlinien, sie stellen also einen optimalen Ausgangspunkt für Ansätze zur Verbesserung der Prävention von apoplektischen Insulten und anderen Komplikationen von Gefäßerkrankungen dar. Allerdings bedarf dieser Prozess auch der Unterstützung von Klinikern, politisch Verantwortlichen, Kostenträgern und Weiterbildungseinrichtungen in der Gesundheitsversorgung sowie von der Öffentlichkeit.

## Introduction

What clinicians and patients need to know are the probabilities of obtaining certain outcomes from the different treatment options in the care environments available to them. For this they need evidence, quality information from research which is local, scientifically robust, current and matches as closely as possible to the clinical status of patients. The problem is that international guidelines, and the medical community in general, inappropriately rank results of randomized trials (or meta-analyses of randomized trials) as superior to all other research results with respect to determining a patient’s best treatment strategy. In the case of carotid artery stenosis management, this scientifically flawed view is a major reason why the field has become so biased towards using ineffective and harmful procedures and has lost track of what is best for patients.

## Randomization and bias

In all the international guidelines on carotid stenosis we have reviewed (A Systematic Review of Guidelines for the Management of Asymptomatic and Symptomatic Carotid Stenosis:  Abbott et al. manuscript in preparation), it is implied or explicitly stated that randomized trial data are the best and most reliable evidence to guide treatment decisions in current, local routine practice. Terms like ‘level A’ or ‘class 1’ are used to indicate the superiority of randomized trial data to the exclusion of non-randomized data. The most important advantage of randomization (according to Wikipedia, 2015, http://en.wikipedia.org/wiki/Randomized_controlled_trial) is that “it minimizes allocation bias, balancing both known and unknown prognostic factors, in the assignment of treatments”; however, randomization cannot always minimize treatment allocation bias. Furthermore, this definition may be misinterpreted as meaning most, if not all, bias can be avoided by randomization; however, experience with carotid stenosis has clearly shown this is incorrect. Bias means “to present or hold a partial perspective and often refusal to even consider the possible merits of alternative points of view” (Wikipedia, 2015, http://en.wikipedia.org/wiki/Bias). Procedural bias with respect to carotid stenosis management has become entrenched and is evident in many ways, as discussed in this article.

## How things should be: a world without procedural bias

If patients’ interests had always come first (and perhaps if we had been smarter) the randomized trials of medical treatment alone versus additional carotid endarterectomy (CEA) for asymptomatic patients (Veterans Affairs Cooperative Study [VACS], Asymptomatic Carotid Atherosclerosis Study [ACAS] and Asymptomatic Carotid Surgery Trial-1 [ACST] [18–21]) would probably not have been done. Instead, we would have done quality non-randomized   measurements of the risk of ipsilateral stroke in patients with asymptomatic carotid stenosis (ACS) given the best available non-invasive medical treatment alone. Medical treatment here refers to the encouragement of a healthy lifestyle and appropriate use of medication. Such measurements did not begin to appear in print until the mid-late 1980s and early 1990s [1], well after these randomized procedural trials were planned and underway. We could have discovered in the 1980s and 1990s that the risk of ipsilateral stroke was lower than expected, probably not warranting the need for additional carotid procedures, especially in a routine practice where procedural outcomes are often not good enough to provide overall benefit or are not measured at all [5].

Even if an average annual ipsilateral stroke rate of approximately 2.3 %, despite medical treatment alone (as in ACAS [18]), was seen as enough to justify CEA trials and then widespread routine practice use, CEA would not have been the standard of care for long. We would have kept measuring the impact of improving medical treatment and discovered much earlier than 2009 [1, 30] or 2013 [2, 3, 29, 40] that stroke rates with medical treatment alone fell below that of CEA patients treated in ACAS from 1995 (as soon as ACAS was published) and have continued to fall [1–3, 29, 30, 40]. In fact, ipsilateral stroke rates are now so low with medical treatment alone ( ≤ 0.5–1.0 % per year) that CEA is more likely to harm than help patients. Even if CEA complication rates were always zero (not possible), CEA is now likely to be an ineffective waste of resources because at least 97.5 % of medically treated patients will not have a stroke due to the carotid lesion during the average 10-year life expectancy from diagnosis [6].

Regardless, we would have been measuring 30-day rates of stroke and death whenever CEA was done and acted to effectively reduce risk of harm when outcomes were below randomized trial standards. In addition, we would not have taken up routine CEA for asymptomatic women or men aged over 75–79 years because evidence of a CEA stroke prevention benefit has been inconclusive or absent [18–20].

In a world without a procedural bias we would *not* have been advising for routine practice CAS for any patient with ACS or symptomatic carotid stenosis (SCS, carotid stenosis and ipsilateral stroke or transient ischemic attack) because CAS causes more strokes and periprocedural deaths (plus/minus periprocedural heart attacks) than CEA [4, 11], particularly in those with ipsilateral stroke or transient ischemic attack (TIA) within the previous 7 days (those most likely to benefit from CEA) [33], women [23, 35] and patients older than 70 years, while a benefit for patients aged < 70 years has not been established [11, 41]. This excessive CAS risk is not to be confused with (or hidden by) non-carotid artery associated causes of disability 1–5 years after CEA or CAS [10]. Furthermore, we would *not* be advising CAS for patients considered at high CEA risk due to vascular anatomy or medical comorbidities because many of these patients have a limited life expectancy (< 3–5 years) and are unlikely to live long enough to benefit, plus a procedural benefit has never been demonstrated in such patients.

In a world without procedural bias we would *not* still be recommending CEA for generally fit but otherwise unselected patients with 50–99 % or 70–99 % SCS and ipsilateral stroke or TIA within the previous 3–6 months as long as the 30-day rate of stroke or death was < 6–7 % (A Systematic Review of Guidelines for the Management of Asymptomatic and Symptomatic Carotid Stenosis, Abbott et al. in preparation). Instead, we would have remeasured the impact of improving medical treatment alone on recurrent stroke rate since symptomatic patients were recruited into the randomized CEA trials 21–34 years ago [7, 17] and found a much lower stroke risk since then. This would have emphasized the need for much more selective CEA and much earlier used CEA as well as better procedural standards for patients with SCS than we have today.

So why have we finished up so far from where we should be, with international guidelines advocating CEA and/or CAS for just about any patients with >50 % carotid stenosis (sometimes for lesser degrees of stenosis [13, 39]) despite contrary or absent evidence (A Systematic Review of Guidelines for the Management of Asymptomatic and Symptomatic Carotid StenosisSystematic review of international carotid management guidelines, Abbott et al. in preparation)? A lot can be learned from an appreciation of biased design and interpretation of randomized carotid procedural trials, as outlined in this article.

## Biased design of randomized trials

### Randomizing to procedures before (or when) knowing the non-procedural risk

One should accurately know the risk of what is to be prevented using the simplest, least risky strategy before embarking on trials of expensive, dangerous and highly operator-dependent procedures. This did not happen for carotid stenosis. We are reinforcing past mistakes with more expensive and slow randomized procedural trials, such as the 'Carotid Revascularization and Medical Management for Asymptomatic Carotid Stenosis Trial'(CREST-2) [25] of relatively unselected ACS patients (similar to those in ACAS and ACST) before measuring the efficacy of current optimal medical treatment alone. However, worse, we now know these patients are most unlikely to have an overall benefit from a carotid procedure. The effectiveness of current optimal medical treatment alone would be most ethically and effectively measured by quality non-randomized trials with built in risk stratification testing and validation [31]. Trials of carotid procedures should only be directed to those with a sufficiently high ipsilateral stroke risk despite current optimal medical treatment alone, if they are reliably identified.

### Randomizing to procedures only

Error was built upon error with the performance of the randomized trials of CEA vs CAS in patients with ACS and SCS which lacked a medical treatment only comparison [4]. These later trials were based on the assumption that medical treatment was simple, largely ineffective and would not change following the randomized trials of CEA versus medical treatment alone, mentioned above. This is an obvious bias in treatment allocation that randomization cannot correct: randomizing patients only to procedures and concluding that a procedure is best.

### Confuse the target population and exaggerate potential procedural benefits

Symptomatic patients have a higher stroke risk with medical treatment alone and are more likely to benefit from CEA than asymptomatic patients [5]. With respect to carotid procedures (which target one artery), ACS is best defined as stenosis in patients with no past history of clinically recognized stroke or TIA in the corresponding arterial territory. This is a distinct and easily identified clinical population. The time by which the recurrent stroke risk in patients with symptomatic carotid stenosis reaches that of ACS is not known, particularly if patients are given current optimal medical treatment; however, results from the North American symptomatic carotid endarterectomy trial (NASCET) suggest it may take at least 2 years [7]. Therefore, inclusion of recently asymptomatic carotid stenosis patients (e.g. asymptomatic for only the last 3–6 months as in some past procedural trials [14, 19, 20]) and extrapolation of results to truly asymptomatic patients will overestimate a potential procedural benefit to the extent that such patients are included and their symptoms are recent.

### Biased use of terminology

Medical treatment is the ongoing diagnosis of vascular disease risk factors and risk reduction by encouraging a healthy lifestyle and appropriate use of medication. It is the best weapon we have to repair arteries and reduce the risk of stroke and other complications of vascular disease. Therefore, it adds to procedural bias when medical treatment is referred to as ‘conservative,’ ‘control’ or ‘natural history’ therapy, as has been common in the past [1, 17, 19]. It is also biased to reserve more effective sounding terminology, such as ‘intervention’, ‘revascularization’ and ‘repair’ to procedures such as CEA or CAS [1, 35, 42].

### Publish as soon as you find a statistically significant result

The 5-year stroke rates with CEA versus medical treatment alone in ACAS and ACST were estimates only, projected using Kaplan-Meier analyses from briefer actual average follow-up durations. The median patient follow-up was just 2.7 years in ACAS [18] and the mean follow-up just 3.4 years in ACST [20]. The assumption that trends will continue adds uncertainty. Significant differences in stroke rates with CEA versus medical treatment alone were not actually measured or observed in ACAS or ACST but based on projections. The results were published as soon as statistical significance (in contrast to clinical significance) was found. The primary outcome comparisons in NASCET [7] and the MRC European carotid surgery trial (ECST) [17] were also 5-year stroke rates and were derived using Kaplan-Meier analyses; however, the mean follow-up durations in these studies more closely matched the primary outcome definitions (5 [7] and 6 [17] years, respectively).

## Bias interpretation of randomized trials

### Assume one or two randomized trials are enough (even if different)

Only one trial involving 825 surgically managed patients (randomized 1988–1993) has shown that CEA may reduce the overall rate of ipsilateral stroke in patients with > 50 % ACS [18]. ACST is the only other randomized trial used to support this CEA stroke prevention benefit [19, 20]. The ACST was different to ACAS with respect to target patient population, randomized treatment arms and outcome measures; nevertheless, these studies have been the basis of guideline recommendations for CEA (and subsequently CAS) for 50–99 % ACS in any place or time since the mid-1990s. However, despite continuing guideline adherence to these trial results, clearly they have not been enough to determine best practice since ACAS and ACST results were often not replicated in routine practice (highlighting the importance of quality local registry data) [5, 24] and they were outdated long ago due to improvements in medical and surgical outcomes [1–3, 28–30, 40]. For the same reasons, clearly NASECT and ECST have not been enough to determine best practice for SCS patients. Quality local registry data and repeated measures of outcomes with medical treatment alone plus/minus additional procedures are required.

### Confuse safety and efficacy outcomes

The CREST-1 trial [14] has been the main driver of recent recommendations in some guidelines that CAS is a suitable (even equivalent [32]) alternative to CEA in patients with ACS and/or SCS. In CREST-1, among all 2502 mixed symptomatic and asymptomatic patients, CAS was not as effective as CEA because it caused nearly twice as many strokes as CEA within the 30-day post-procedural period with a hazard ratio (HR) of 1.8 and 95 % confidence interval (CI) of 1.1–2.8 (*P* = 0.01). The first 30 days following a procedure is the period of highest risk for procedure-associated adverse events and where significant treatment differences most often appear. In CREST-1 the CAS-related stroke excess was still evident in 4 years at study end (HR 1.4, CI 1.0–2.1, *P* = 0.049), particularly if periprocedural death was included (HR 1.5, CI 1.1–2.2, *P* = 0.03) [14]; however, in CREST-1 myocardial infarction (an adverse or safety outcome) was used in a balancing act to show no ‘statistically’ significant difference between CAS and CEA in the chosen primary outcome measure (30-day periprocedural stroke, death or myocardial infarction or any ipsilateral stroke within 4 years of randomization) [14]. As well documented, in CREST-1, among all 2502 mixed symptomatic and asymptomatic patients, periprocedural clinically defined myocardial infarction was twice as common with CEA than CAS (28 versus 14 myocardial infarctions) [9, 14]. However, periprocedural stroke was nearly twice (1.9 times) as common as periprocedural clinically defined myocardial infarction (81 strokes versus 42 myocardial infarctions) and approximately 1.3 times as common as myocardial infarction defined clinically or with biomarker change only (81 strokes versus 62 myocardial infarctions) [9, 14]. Because periprocedural stroke was more common than periprocedural myocardial infarction in CREST-1, CAS still caused more of these clinically defined adverse outcomes than CEA (66 versus 57), although this did not reach statistical significance (HR 1.2, 95 % CI 0.8–1.7, *P* = 0.38) [14]. The incidence of myocardial infarction beyond the 30-day periprocedural period was not reported [9, 14].

In randomized trials of CAS versus CEA, where both 30-day periprocedural outcomes were reported, stroke (most caused by CAS) was overall approximately 4.5 times as common as periprocedural clinically defined myocardial infarction [12, 14–16, 22, 26]. A meta-analysis of all available randomized trials of CAS versus CEA (providing larger patient numbers than in CREST-1 alone) demonstrated among symptomatic patients a statistically significant higher risk of 30-day periprocedural stroke, death or myocardial infarction with CAS (odds ratio, OR 1.44, 95 % CI 1.15–1.80, *P* = 0.002) [11], while the rate of ipsilateral stroke after the periprocedural period did not differ between treatments (OR 0.93, 95 % CI 0.60–1.45, *P* = 0.76) [11].

No randomized trial has been sufficiently powered to test for a difference in rate of stroke with or without myocardial infarction in ACS patients alone; however, the direction of effect in CREST-1 (largest relevant randomized trial) was indicative of approximately double the rate with CAS (HR 1.86, 95 % CI 0.95–3.66, *P* = 0.07), similar to that of SCS patients [14]. Adverse outcomes, such as myocardial infarction are a safety indicator and should not be confused with efficacy indicators because this can camouflage treatment differences and add to an inappropriate procedural bias, as has occurred with the reporting of CREST-1 and other studies [26, 42].

### Assume randomized trial results apply to all patients

Using ACAS and ACST results, CEA reduced the overall average annual stroke rate by 0.5–1.0 % [18–20] despite a 30-day stroke or death rate of about 2.5 %. However, as mentioned, a benefit for women was not seen in ACAS [18] or ACST at 5 years [20] and was of borderline statistical significance in women younger than 75 years in ACST at 10 years [19]. Furthermore, a CEA benefit for men with ACS aged over 75–79 years or women over 75 years has not been established [18–20]. Using the combined results of the randomized trials of CEA versus medical treatment alone in patients with > 70 % SCS, CEA reduced the average annual rate of ipsilateral stroke by 3% despite a 30-day stroke or death rate of approximately 7 % [36]. However, those men with index symptoms within the previous 2 weeks, those with a life expectancy > 3 years and patients over 75 years were most likely to benefit [36–38]. Furthermore, there is no randomized trial evidence that CAS benefits any patients with ACS or SCS, only evidence of overall harm; however, international guidelines often endorse CEA and/or CAS for just about all patients with carotid stenosis over 50–70 %, instead of limiting endorsements to subgroups shown to benefit (Systematic Review of Guidelines for the Management of Asymptomatic and Symptomatic Carotid Stenosis, Abbott et al. in preparation).

### Discount non-randomized data

Randomized trials are not always possible or the most appropriate way to address a clinical question. They may even be unethical, as indicated previously. Quality non-randomized trial data (including from quality registries) should determine the need for randomized trials and ensure favored randomized trial results are at least as good in routine practice. Non-randomized studies, therefore, should be the ‘work-horse’ of the evidence base, reserving randomized studies for situations where there is real uncertainty and reasonable comparisons to be made. If non-randomized study data are considered inferior, the goal is to improve rather than dismiss them [6].

Contemporary guidelines place far too much emphasis on the presence of randomization and not enough on other relevant issues, such as how well the study sample is described and how relevant it is to the clinical question; whether or not target populations are adequately defined or all reasonable treatment strategies were compared and described adequately; whether or not treatment strategies were current, optimal and adhered to; whether or not the main outcome measure was related to efficacy or safety and if it was appropriate for the clinical question; whether or not any treatment differences were clinically meaningful (rather than statistically significant); whether the study methods were practical or can be replicated in routine practice, whether or not results have been adequately validated and the extent to which investigator income was dependent on the study results. Guideline writers need to recognize the complimentary power of randomized and non-randomized data and the limitations they may share.

## Conclusions

Randomized trials are a subset of prospective, observational cohort studies in which patients are ‘randomized’ to two or more possible treatment strategies. As explained previously, they are subject to the same limitations as non-randomized trials, including bias in patient selection, treatment allocation and result interpretation. Randomization cannot correct for all these potential problems and is not usually the best approach to answer a clinical question. The relative dismissal of medical treatment in the management of carotid stenosis and bias in favor of unhelpful procedures has occurred because of overreliance on the relevance of randomized trial data. There is an urgent need for guideline writers to address this and update and otherwise improve their recommendations.

In addition, there are likely to be wider influences that encourage a procedural bias in carotid stenosis management, such as the way medical services are funded. A procedural bias may be encouraged where there is a proportionally higher reimbursement for time performing procedures rather than spent in valuable communication with patients and others to facilitate accurate diagnosis and best treatment. Furthermore, medical funding has been ‘activity’ rather than patient outcome focused for too long. Funders of health services (including especially governments) need to become more responsible and fund what works to improve patient outcomes, stop paying for what does not and fund the research to tell the difference.

There is a tremendous need for clinicians everywhere to systematically measure key outcomes of the treatment they give patients, whether or not it is procedural. This could be much more easily achieved if doctors’ records were electronic and standardized with respect to topics covered and crucial terminology used so that the information could be used for two purposes: immediate patient care (medical reports generation) and care improvement through health services research (registries) [6]. Doctors who don’t count, don’t count [8]. The situation is urgent with government spending on health services growing unsustainably [27, 34], while salaries for even the ‘best’ independent international clinical researchers are nearly impossible to secure, even for a short time. Finally, there is a great need to educate children before they leave school about how to recognize complications of highly preventable conditions, such as atherosclerosis and understand the great effectiveness of them adopting a healthy lifestyle and appropriate use of medication.
